# Effectiveness of Computerized Cognitive Remediation Therapy on Executive Functions and Clinical Symptoms in Children and Adolescents With ADHD: A Meta-Analysis of Randomized Controlled Trials

**DOI:** 10.31083/AP39972

**Published:** 2026-03-17

**Authors:** Junrong Ye, Yuhan Zhang, Na Ma, Tian Zhou, Lexin Yuan, Yanheng Wei, Jinrong Li, Xueyu Zheng, Dingjie Liu, Jianxiong Guo, Aixiang Xiao

**Affiliations:** ^1^Department of Nursing Administration, Department of Social Psychiatry, The Affiliated Brain Hospital, Guangzhou Medical University, 510370 Guangzhou, Guangdong, China; ^2^Guangdong Engineering Technology Research Center for Translational Medicine of Mental Disorders, 510370 Guangzhou, Guangdong, China; ^3^School of Nursing, Guangzhou Medical University, 510475 Guangzhou, Guangdong, China; ^4^Department of Geriatric Neurology, Department of Social Psychiatry, The Affiliated Brain Hospital, Guangzhou Medical University, 510370 Guangzhou, Guangdong, China; ^5^Department of Traditional Chinese Medicine, Department of Social Psychiatry, The Affiliated Brain Hospital, Guangzhou Medical University, 510370 Guangzhou, Guangdong, China

**Keywords:** attention deficit disorder with hyperactivity, computer-assisted therapy, cognitive remediation, executive functions, clinical symptoms, meta-analysis

## Abstract

**Background:**

This meta-analysis of randomized clinical trials (RCTs) aimed to determine whether computer-assisted cognitive remediation therapy (CCRT) is a feasible adjunctive treatment to improve executive function and clinical symptoms in adolescents and children with attention deficit hyperactivity disorder (ADHD).

**Methods:**

We systematically searched 9 English and Chinese databases from database inception until May 2025. Randomized controlled trials investigating CCRT in children and adolescents with ADHD were collected. The risk of bias was assessed using the Cochrane Collaboration's tool for assessing risk of bias (RoB 2.0) and the Jadad Scale. Standardized mean differences in post-intervention data were calculated using RevMan 5.4 software. This study adhered to the Preferred Reporting Items for Systematic Reviews and Meta-Analyses (PRISMA) guidelines and was registered on the International Prospective Register of Systematic Reviews (PROSPERO) platform.

**Results:**

This review included 17 RCTs comprising 1087 cases. In the CCRT intervention groups, there was a significant decrease in overall executive function (standardized mean difference (SMD) = –0.23; 95% CI = [–0.45, –0.01]; *p* = 0.04), working memory (SMD = –0.25; 95% CI = [–0.46, –0.03]; *p* = 0.03), inhibition (SMD = –0.25; 95% CI = [–0.44, –0.05]; *p* = 0.01), planning (SMD = –0.26; 95% CI = [–0.50, –0.01]; *p* = 0.04), and inattention (SMD = –0.22; 95% CI = [–0.39, –0.05]; *p* = 0.01) compared to the control groups; however, results did not show significant effects on cognitive flexibility, overall clinical symptoms, or hyperactivity/impulsivity compared to the control group.

**Conclusion:**

CCRT played an important role in improving specific executive functions and attention symptoms in children and adolescents with ADHD. However, high-quality research is needed to validate these preliminary findings.

**The PROSPERO Registration:**

CRD42024619958, https://www.crd.york.ac.uk/PROSPERO/view/CRD42024619958.

## Main Points

1. Computerized Cognitive Remediation Therapy (CCRT) significantly improved overall executive functions in children and adolescents diagnosed with Attention-Deficit/Hyperactivity Disorder (ADHD).

2. Regarding executive function sub-dimensions, CCRT effectively improved working memory, inhibitory control, and planning in adolescents and children with ADHD, without significant effects on cognitive flexibility and emotional control.

3. CCRT effectively reduced inattention symptoms but did not significantly improve overall ADHD clinical symptoms or hyperactivity/impulsivity.

## 1. Introduction

Attention deficit hyperactivity disorder (ADHD) is one of the most common neurodevelopmental disorders among minors, affecting 7.6% of children and 5.6% of adolescents, with an increasing trend [1, 2]. The clinical symptoms of ADHD, such as inattention and hyperactive impulsivity not only impair patients’ learning capacity but also have a profound negative impact on social function and family life [3, 4]. Empirical studies have shown that the neural mechanism underlying the above dysfunctions may be closely linked to developmental irregularities in executive functions [5].

As a core component of advanced human cognitive ability, executive functions encompass multiple core components that collectively underpin higher-order cognitive processes essential for adaptive behavior, including working memory, inhibitory control, cognitive flexibility, goal-directed planning, and problem-solving strategies [5]. Research indicates that individuals with ADHD exhibit significant neurodevelopmental abnormalities [6]. Notably, patients with ADHD demonstrated a 25% increase in inhibitory response latency and twice the error rate compared to neurotypical peers during standardized Stop Signal Task (SST) assessments [7]. Besides, the working memory capacity of ADHD patients was significantly reduced compared to typically developing peers, with deficits of 15% in their verbal working memory tasks and 10%–15% deficits in visual-spatial working memory tasks. Information refresh efficiency, indexed by delayed response tasks, revealed a 200–300 ms temporal delay effect in updating task-relevant information [8, 9, 10].

The multidimensional impairments in executive functions and clinical symptoms among individuals with ADHD are associated with marked difficulties in handling complex tasks. A cohort study conducted in northern Finland showed that adolescents with ADHD exhibited significantly lower academic performance at age 16 than their non-ADHD peers, with an average subject score of 6.63 (compared with 7.61 out of 10 for the control group) [11]. The performance disparity was particularly pronounced in subjects requiring high executive functions such as mathematics and reading, and further widened with increasing academic complexity [12, 13]. Notably, executive function deficits and clinical symptoms exhibited significant longitudinal stability, with persistent deficits in adulthood associated with a range of vocational outcomes including failure to meet self-standards and unfulfilled vocational potential, elevated absenteeism, reduced occupational attainment, diminished job tenure, and higher unemployment rates [14, 15, 16].

Clinically, medication is primarily employed to alleviate executive function deficits and core symptoms of ADHD [17], often supplemented with behavioral or psychological interventions to optimize overall therapeutic efficacy [18]. However, pharmacological treatment, such as methylphenidate and atomoxetine, is associated with adverse outcomes. Among children and adolescents treated with methylphenidate, 67.3% experienced side effects, including decreased appetite (34%), nausea (10.9%), depression (21%), and insomnia (13%), with 3.6% of patients discontinuing treatment due to serious adverse reactions [19]. Atomoxetine use was associated with higher rates of irritability (22.6%), mood swings (17.3%), and suicide-related signals (reporting odds ratio (ROR) = 10.8) compared to other medications [20]. Standard pharmacological treatments have demonstrated cardiovascular implications, with methylphenidate associated with increased heart rate, while atomoxetine has been linked to rare but serious complications such as prolonged QT interval and cardiogenic shock [19, 20, 21].

Traditional medication therapies have shown limited efficacy in treating neurocognitive deficits, prompting the development of non-pharmacological interventions focusing on executive function networks. Among these, Computerized Cognitive Remediation Therapy (CCRT) has emerged as a promising alternative therapy. CCRT is based on the theory of neuroplasticity and aims to strengthen the functional connectivity of the prefrontal-parietal neural network [21]. Compared to traditional cognitive training, CCRT leverages digital platforms such as computer programs, tablets or smartphones to deliver cognitive exercises that target skills such as attention, memory, executive functions, and inhibitory control [22], thereby addressing the need for accessible concerns.

Besides, CCRT could dynamically adjust the training difficulty in real-time to align with children’s abilities, providing personalized training that maintains continuous challenge and engagement [23], thereby improving cognitive gains and long-term skill retention, especially in areas such as attention, working memory [24], and executive functions [25]. Patients also exhibit high levels of attention and motivation in CCRT due to the game-like design in pediatric programs, which enhances engagement and sustains focus, directly addressing the fundamental difficulties related to task persistence in children with ADHD [26].

Current evidence suggests that CCRT is a promising intervention for ADHD patients across all age ranges. However, few studies on the efficacy of CCRT have focused on its effectiveness in children and adolescents [27, 28]. Although CCRT has been widely applied in children and adolescents with ADHD, inconsistent results have been reported on core dimensions of executive functions (such as cognitive flexibility and inhibitory control) and improvement of clinical symptoms in children and adolescents [22, 29, 30]. In addition, a study published in 2019 indicated no inter-group difference in working memory, attention and executive function between basic non-adaptive and adaptive CCRT interventions [31]. However, most meta-analyses have used non-adaptive CCRT in the control group for pooled effect estimation [28] and this methodological bias has systematically underestimated the true therapeutic effect of CCRT.

Therefore, this study proposes to systematically evaluate the improvement of executive functions and clinical symptoms in adolescents and children with ADHD, excluding studies in which the control group received non-adaptive CCRT to recalculate the actual effect of CCRT on adolescents and children with ADHD. This study aimed to provide evidence for the clinical application of CCRT in treating children/adolescents with ADHD.

## 2. Method

The proposed meta-analysis was registered with the International Prospective Register of Systematic Reviews (PROSPERO) (CRD42024619958) and adhered to the Preferred Reporting Items for Systematic Reviews and Meta-Analyses (PRISMA 2020) guidelines [32]. For PRISMA 2020 checklist, see **Supplementary Material**.

### 2.1 Search Strategy

We systematically searched nine databases: PubMed (https://pubmed.ncbi.nlm.nih.gov), Web of Science (https://www.webofscience.com), Embase (https://www.embase.com), Cochrane Library (https://www.cochranelibrary.com), PsycINFO (https://www.ebsco.com/products/research-databases/apa-psycinfo), China National Knowledge Infrastructure (CNKI, https://www.cnki.net), China Biology Medicine Disc (CBM, https://www.sinomed.ac.cn/zh/index.jsp), Chinese Scientific Journal Database (VIP, https://qikan.cqvip.com/), and WANFANG (https://www.wanfangdata.com.cn) for the literature from inception to May 25, 2025. The search utilized a blend of subject and free terms, detailed search strategy in the **Supplementary Material**.

### 2.2 Selection Criteria

The criteria for inclusion in our meta-analysis were established using the PICOS (Population, Intervention, Comparison, Outcomes, Study Design) framework: Participants: patients under 18 years old diagnosed with ADHD by Diagnostic and Statistical Manual of Mental Disorders, Third Edition/International Classification of Diseases, Ninth Revision (DSM-III/ICD-9) onwards, or who scored above the cut-off on validated ADHD rating scales, regardless of the presence of common comorbidities. Intervention: Eligible interventions encompassed all CCRT modalities. Comparison: no treatment (wait-list), placebo (pill and psychological placebo), or treatment as usual (TAU). Treatment as usual may have involved diagnostic cognitive assessments, psycho-education, pedagogical counseling, questionnaires for parents and teachers, home and school visits and medical treatment. Outcomes: the primary outcomes were overall executive function and clinical symptoms as quantified by questionnaires or neuropsychological tests. Secondary outcomes were the sub-dimensions of executive functions and ADHD symptoms. Study design: randomized controlled trials published in peer-reviewed journals. Studies were required to demonstrate no significant difference in age, gender, condition, etc. of the study subjects, ensuring good and comparable baseline consistency.

The exclusion criteria were as follows: (1) patients with comorbid autism, tic disorder, epilepsy, or other mental disorders; (2) studies where CCRT was used in the control group; (3) studies where full data is not available; (4) republished studies; (5) studies containing incomplete data or data that cannot be transformed for analytical purposes; (6) articles not published in Chinese or English.

### 2.3 Study Selection

All identified studies were imported into EndNote X9 (Clarivate Analytics, London, UK) Duplicates were removed, followed by screening of titles, abstracts, and full texts, in line with the meta-analysis selection criteria. Discrepancies between the two independent researchers were resolved by a third researcher.

### 2.4 Data Extraction

Data extraction was independently conducted by two researchers, following the eligibility criteria, with any conflicts resolved by consulting a third researcher. The extracted information included: (1) title, author, year and setting; (2) participant demographics, including gender, age and number of participants; (3) intervention details: name, type and content; (4) control group methodology and specific description of the control group; (5) outcome measures: including means and standard deviations for executive functions and ADHD symptoms at the first point post-intervention. If studies reported multiple executive function outcomes, we prioritized the results reported on the Behavior Rating Inventory of Executive Function (BRIEF) scales, given their established good validity and reliability [33], followed by results from commonly used neuropsychological tests. For both teacher and parent evaluations, we selected the stricter blind outcomes based on the experimental site.

### 2.5 Quality Assessment

The risk of bias in each study was independently evaluated by two reviewers using the Risk of Bias (RoB) 2.0 (Cochrane Collaboration: https://www.cochrane.org/handbook/current/chapter-08#section-8-5) tool and the Jadad Scale [34, 35]. The RoB 2.0 tool assessed each RCT across 5 domains: selection bias, performance bias, detection bias, attrition bias, and reporting bias. The risk of bias was categorized into three levels: low, unclear, and high. Randomized controlled trials scoring ≥3 on the Jadad scale were classified as high quality. Quality assessments of primary and secondary outcome findings were conducted independently using the Grading of Recommendations, Assessment, Development, and Evaluation (GRADE) system [36]. Conflicting results were resolved through discussion among the three reviewers.

### 2.6 Data Analysis

The data synthesis process was conducted utilizing a random-effects model according to Cochrane Collaboration guideline [37]. Data analysis was performed in RevMan 5.4 (The Cochrane Collaboration, London, UK), calculating the standardized mean difference (SMD) with a 95% confidence interval (CI) to estimate effect sizes based on endpoint scores. Studies were assessed for heterogeneity using Cochrane’s Q and *I*^2^ tests. Significant heterogeneity was denoted by *p* < 0.1 or *I*^2^ ≥50% [38]. Sensitivity analysis was performed by sequentially excluding each study to evaluate the robustness and stability of the results. Publication bias analysis for the primary outcome was conducted using funnel plots and an Egger’s test when more than 10 articles were included, with a significance threshold of 0.05 based on 2-tailed *p* values [39]. All data analyses were conducted using STATA Version 17 (StataCorp LLC, College Station, TX, USA).

## 3. Results

### 3.1 Search Results

**Fig. 1.**
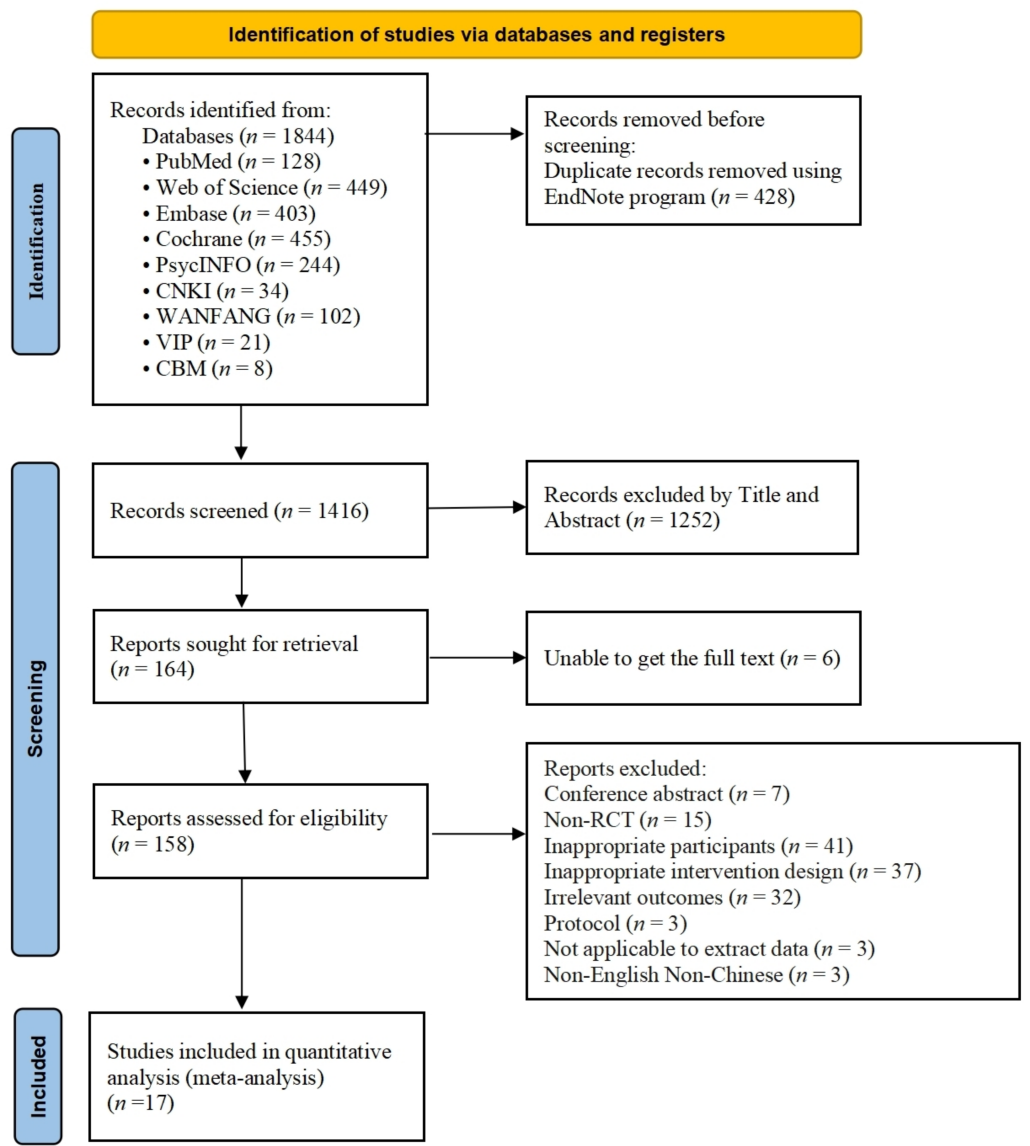
**PRISMA flow diagram of study selection**. PRISMA, Preferred Reporting Items for Systematic Reviews and Meta-Analyses; RCT, randomized controlled trial.

Fig. 1 illustrates the meta-analysis workflow. A total of 1844 studies were initially retrieved from databases, including PubMed (*n* = 128), Web of Science (*n* = 449), Embase (*n* = 403), The Cochrane Library (*n* = 455), PsycINFO (*n* = 244), CNKI (*n* = 34), WANFANG (*n* = 102), VIP (*n* = 21) and CBM (*n* = 8). After removing duplicates (*n* = 428), 1252 studies were excluded based on title and abstract. Subsequently, 6 studies were eliminated due to unavailability of the full text, leaving 158 eligible studies. Upon full review of these 158 studies, 141 were excluded due to the following reasons: conference abstract, non-RCT, inappropriate participants, inappropriate intervention design, irrelevant outcomes, protocol, not applicable to extract data, or being published in a language other than English or Chinese. Finally, 17 studies were included [40, 41, 42, 43, 44, 45, 46, 47, 48, 49, 50, 51, 52, 53, 54, 55, 56].

### 3.2 Study Characteristics

17 RCTs, comprising 1087 patients, were selected for this study, with participant ages ranging from 7.60 ± 0.93 to 15.6 ± 0.99. Among these RCTs, the most common control group was placebo control (*n* = 7), followed by TAU (*n* = 5) and wait-list (*n* = 5). The setting for conducting CCRT was not reported in one trial, and the remaining 16 trials were conducted across five settings: home (*n* = 6), school (*n* = 3), research institution (*n* = 3), clinic/hospital (*n* = 2), and a mixed setting (i.e., home, lab, library, clinic, school; *n* = 2). For an in-depth exploration of the included studies, please refer to Table 1 (Ref. [40, 41, 42, 43, 44, 45, 46, 47, 48, 49, 50, 51, 52, 53, 54, 55, 56]), which provides a detailed description of the participant samples.

**Table 1.** **Characteristics of the included studies**.

Author, year	Age (yrs; M ± SD)	Participant (E: W/M; C: W/M)	Intervention				Control	Outcomes	Measures	Jadad
			Type	Length	Duration, frequency	Setting				
Bikic *et al*., 2017 [40]	15.6 ± 0.99	E: 2/7; C: 2/6	SBT program (a-CCRT)	7 weeks	① 30 min/session	Home	Tetris (Placebo)	① Overall clinical symptoms	① ADHD-RS-T	5
					② 5 sessions/week					
Bikic *et al*., 2018 [41]	9.95 ± 1.7	E: 6/29; C: 5/30	ACTIVATE™ (na-CCRT)	8 weeks	① no report	Home	TAU	① Overall executive functions	① BRIE-T	2
					② 6 sessions/week			② Working Memory	② ADHD-RS-T	
								③ Inhibition		
								④ Cognitive Flexibility		
								⑤ Planning		
								⑥ Emotional Control		
								⑦ Overall clinical symptoms		
								⑧ Inattention		
								⑨ Hyperactivity/Impulsivity		
Bioulac *et al*., 2020 [42]	8.9 ± 1.2	E: 2/14; C: 8/11	Virtual cognitive remediation (a-CCRT)	6 weeks	① 30 min/session	Clinic	Psychotherapy placebo training (Placebo)	① Overall clinical symptoms	ADHD-RS-P	3
					② 2 sessions/week			② Inattention		
								③ Hyperactivity/Impulsivity		
de Oliveira Rosa *et al*., 2021 [43]	10.66 ± 1.79	E: 11/13; C: 7/14	ACTIVATE™ (na-CCRT)	12 weeks	① 30 min/session	Lab at school/hospital	Placebo cognitive training (Placebo)	① Overall executive functions	① Working Memory Test	3
					② 4 sessions/week			② Working Memory	② Go/No-Go Test	
								③ Inhibition		
Kim *et al*., 2022 [44]	9.10 ± 1.78	E: 3/12; C: 4/11	NeuroWorld DTx digital treatment (a-CCRT)	4 weeks	① 30 min/session	Research institution	As usual medication treatment (TAU)	① Overall executive functions	① Flanck task	3
					② no report			② Inhibition	② K-ARS-P	
								③ Overall clinical symptoms		
								④ Inattention		
								⑤ Hyperactivity/Impulsivity		
Kirk *et al*., 2024 [45]	7.60 ± 0.93	E: 7/21; C: 10/17	Tali Train Program (a-CCRT)	5 weeks	① 20 min/session;	Home	Placebo control program (Placebo)	① Overall executive functions	① BRIEF-2-P	5
					② 5 sessions/week			② Overall clinical symptoms	② SWAN-P	
Medina *et al*., 2021 [46]	9.45 ± 1.29	E: 2/13; C: 2/12	KAD_SCL_01 games (a-CCRT)	12 weeks	① 15–20 min/session;	Home	Video games (Placebo)	① Overall executive functions	① BRIEF-P	4
					② 3 sessions/week			② Working Memory	② EDAH scales-P	
								③ Inhibition		
								④ Cognitive Flexibility		
								⑤ Emotional Control		
								⑥ Planning		
								⑦ Overall clinical symptoms		
								⑧ Inattention		
								⑨ Hyperactivity/Impulsivity		
van der Oord *et al*., 2014 [47]	9.75 ± 1.28	E: 2/16; C: 5/17	Braingame Brian (a-CCRT)	5 weeks	① 40 min/session;	Home	Wait-list	① Overall executive functions	① BRIEF-P	2
					② 5 sessions/week			② Working Memory	② DBDRS-T	
								③ Inhibition		
								④ Cognitive Flexibility		
								⑤ Overall clinical symptoms		
								⑥ Inattention		
								⑦ Hyperactivity/Impulsivity		
Kollins *et al*., 2020 [48]	9.65 ± 1.30	E: 55/125; C: 45/123	AKL-T01 (a-CCRT)	4 weeks	① 25 min/day;	Research institution	A digital control (Placebo)	① Overall clinical symptoms	① BRIEF-P	5
					② 5 days/week			② Inattention	② ADHD-RS-P	
								③ Hyperactivity/Impulsivity		
Sol Sandberg and McAuley, 2022 [49]	11.59 ± 2.17	E: 2/18; C: 6/14	MCT (a-CCRT)	10 weeks	① 30 min/session;	Hospital	TAU	① Overall executive functions	① BRIEF-2-P	3
					② 3 sessions/week			② Working Memory	② AWMA spatial processing	
								③ Overall clinical symptoms	③ ASEBA-T	
Steiner *et al*., 2011 [51]	12.4 ± 0.9	E: 13; C: 15	SCF (a-CCRT)	16 weeks	① 45 min/session;	School	Wait-list	① Overall executive functions	① BRIEF-P	3
					② 2 sessions/week			② Overall clinical symptoms	② CRS-R-P	
								③ Inattention		
								④ Hyperactivity/Impulsivity		
Steiner *et al*., 2014 [50]	8.65 ± 1.05	E: 11/23; C: 11/25	CCT (a-CCRT)	20 weeks	① 45 min/session;	School	Wait-list	① Overall executive functions	① BRIEF-P	3
					② 3 sessions/week			② Working Memory	② Conners 3-P	
								③ Inhibition		
								④ Emotional Control		
								⑤ Planning		
								⑥ Overall clinical symptoms		
								⑦ Inattention		
								⑧ Hyperactivity/Impulsivity		
Liao *et al*., 2022 [52]	9.95 ± 1.77	E: 3/22; C: 6/19	WWNF (a-CCRT)	10 weeks	① 20 hours	no report	Wait-list	① Overall executive functions	① Go/No-Go tasks	3
								② Inhibition	② ToL	
								③ Planning	③ WCST—Pers-Errors-p	
								④ Cognitive Flexibility	④ daily EF questionnaire	
								⑤ Overall clinical symptoms	⑤ SNAP-IV-P	
								⑥ Inattention		
								⑦ Hyperactivity/Impulsivity		
Egeland *et al*., 2013 [56]	10.4 ± 0.7	E: 33; C: 34	cogmed’s RoboMemo program (a-CCRT)	5–7 weeks	① 30–45 min/session	School	TAU	① Overall executive functions	① BVRT	3
					② no report			② Working Memory	② ARS-IV-P	
								③ Overall clinical symptoms	③ BRIEF-P	
								④ Inattention		
								⑤ Hyperactivity-Impulsivity		
Jones *et al*., 2020 [53]	10.14 ± 2.02	E: 13/28; C: 12/27	N-Back Cognitive Training (a-CCRT)	5 weeks	① no report	Home and lab/library/public place	A general knowledge and vocabulary task	① Overall executive functions	① BRIEF-P	2
					② 4 sessions/week		(Placebo)	② Working Memory	② CPRS-R-P	
								③ Inhibition		
								④ Emotional Control		
								⑤ Planning		
								⑥ Initiation		
								⑦ Overall clinical symptoms		
								⑧ Inattention		
								⑨ Hyperactivity-Impulsivity		
Kim and Lee, 2025 [55]	8.57 ± 1.58	E: 3/13; C: 2/12	NeuroWorld DTx (a-CCRT)	4 weeks	① 25 min/session	Home	TAU	① Overall clinical symptoms	K-ARS-P	5
					② 5 sessions/week			② Inattention		
								③ Hyperactivity/Impulsivity		
Trinczer and Shalev, 2024 [54]	10.46 ± 1.10	E: 13/16; C: 7/17	Set game (na-CCRT)	9 weeks	① 75 min/session	Research institution	Wait-list	① Working Memory	① BRIEF	5
					② 2 sessions/week			② Inhibition	② CBCL-APS	
								③ Emotional Control		
								④ Planning		
								⑤ Inattention		

BRIEF-P, Behavioral Rating Inventory of Executive Function-Parent Report Form; BRIEF-T, Behavioral Rating Inventory of Executive Function-Teacher Report Form; BRIEF-2-P, Behavior Rating Inventory of Executive Function Second Edition-Parent Report Form; AWMA, Automated Working Memory Assessment; ToL, The Tower of London; WCST, Wisconsin Card Sorting Task; Pers-Errors-p, percent-perseveration errors; BVRT, Benton Visual Retention Test; ADHD-RS-P, Attention-Deficit/Hyperactivity Disorder Rating Scale-Parent Report Form; ADHD-RS-T, Attention-Deficit/Hyperactivity Disorder Rating Scale-Teacher Report Form; K-ARS-P, Korean ADHD Rating Scale-Parent Report Form; SWAN-P, Strengths and Weaknesses of ADHD symptoms and Normal behavior scale-Parent Report Form; EDAH-P, Evaluación delTrastorno por Deficit de Atención e Hiperactividad-Parent Report Form; DBDRS-T, Disruptive Behavior Disorder Rating Scale-Teacher Report Form; ASEBA-T, Achenbach System of Empirically Based Assessment-Teacher Report Form; CRS-R-P, Conners’ Rating Scales–Revised-Parent Report Form; Conners 3–P, the Conners 3–Parent Assessment Report; SNAP-IV-P, Swanson, Nolan, and Pelham version IV scale-parent form; ARS-IV-P, ADHD-Rating Scale IV-Parent Report Form; CPRS-R-P, The Conners’ Parent Questionnaire–Revised, Long Form-Parent Report Form; CBCL-APS, Child Behavior Checklist-Attention Problems Subscale; SBT, Scientific Brain Training; MCT, Modified Cogmed Treatment; SCF, standard computer format; CCT, computer cognitive training; WWNF, Will Well Neurofeedback; a-CCRT, adaptive CCRT; na-CCRT, non-adaptive CCRT; EF, Executive Function; TAU, treatment as usual; W, women; M, man.

### 3.3 Assessment of Study Quality

**Fig. 2.**
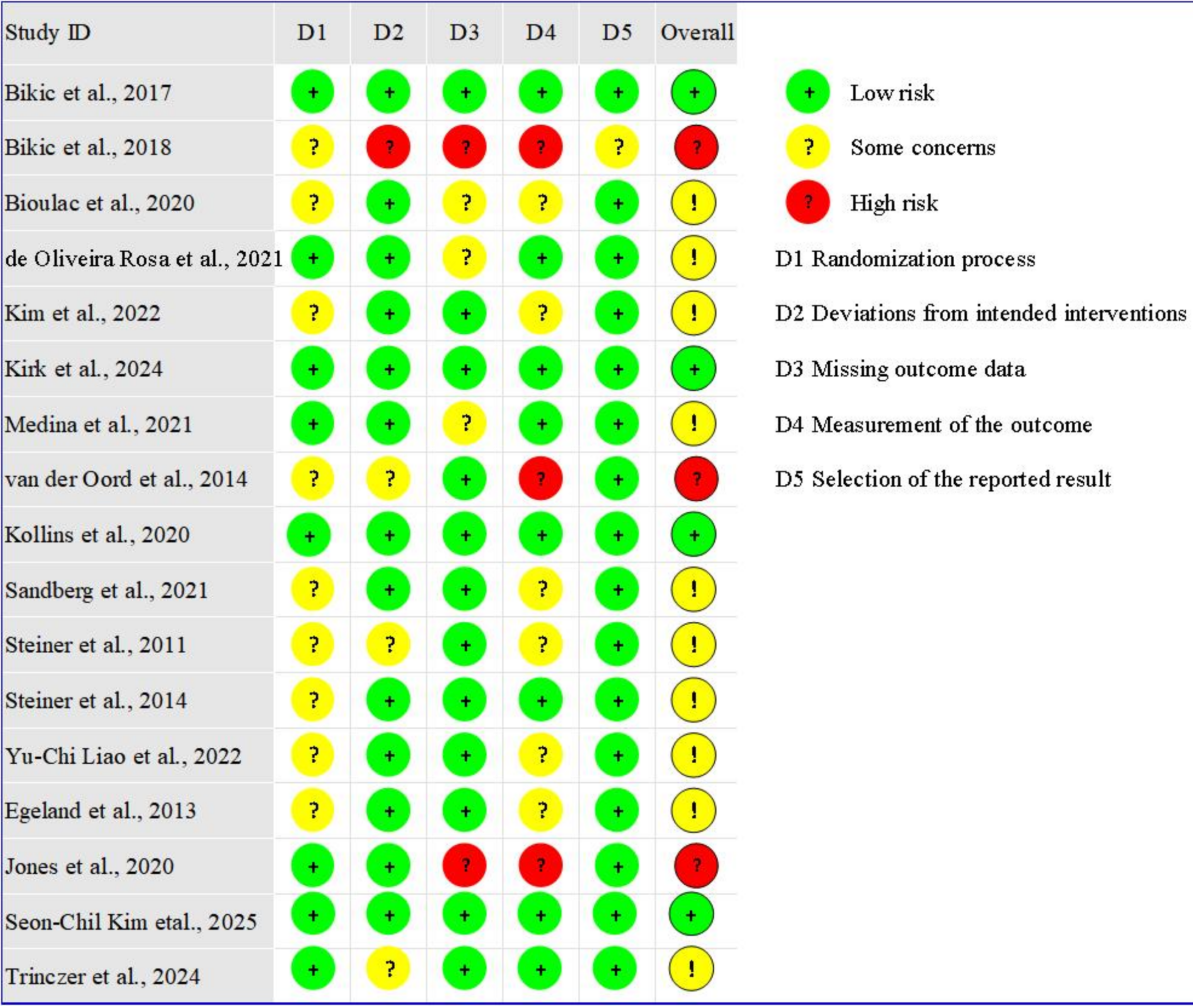
**Risk of bias graph of included studies**.

**Fig. 3.**
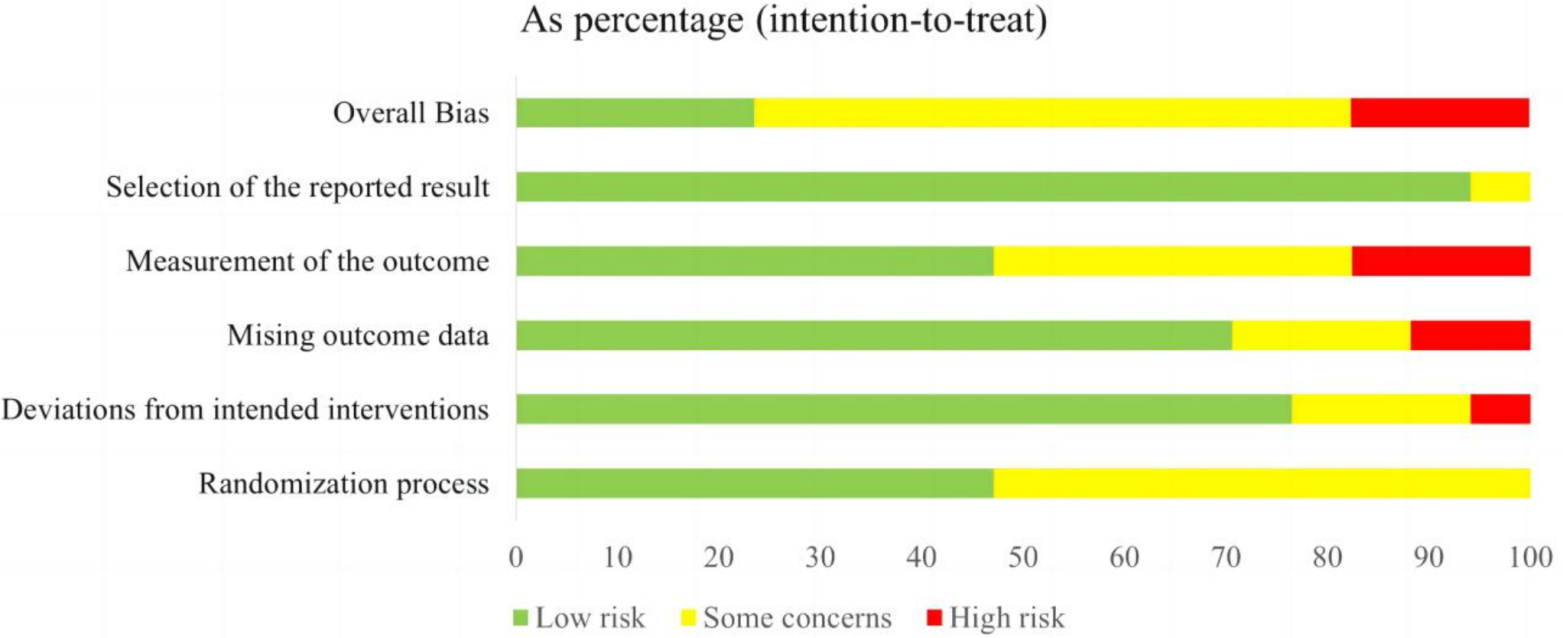
**Risk of bias summary of included studies**.

Figs. 2,3 present the methodological risk of bias assessments for the included 17 studies. All studies reported randomization; however, 9 studies did not mention allocation concealment, possibly leading to selection bias. For deviations from intended interventions, 1 study was rated as high risk due to the absence of blinding. Besides, 2 studies had missing outcome data and were therefore considered to have a high risk of attrition bias. Outcome assessors were blinded in 8 studies, while among the remaining 9 studies, 3 were rated as high-risk and 6 raised concerns regarding bias in outcome measurements. Regarding selective reporting bias, 16 studies were deemed to provide sufficient information for a conclusive judgment and were rated as low-risk. Overall, 4 studies were rated as “low risk of bias”, 10 studies were rated as having “some concerns”, and 3 studies were rated as “high risk of bias”. This was consistent with the Jadad scores (Table 1), which classified three studies as low quality (Jadad scales <3). In accordance with the GRADE criteria, the aggregate evidence grades for the 9 eligible meta-analysis outcomes were categorized as “low” (22.2%, n = 2/9), “moderate” (44.4%, n = 4/9), and “high” (33.3%, n = 3/9). More details are provided in the table GRADE analyses in the **Supplementary Material**.

### 3.4 Outcomes of the Meta-Analysis

#### 3.4.1 Overall Executive Functions

**Fig. 4.**
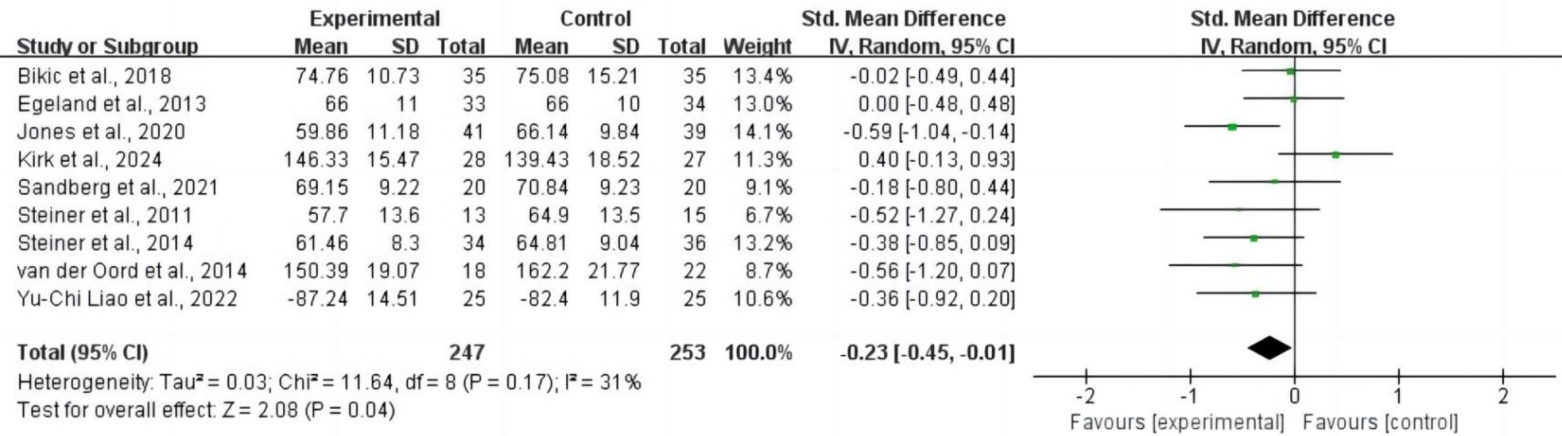
**Overall executive functions**. CI, confidence interval.

For overall executive functions, nine studies comprising 500 subjects provided usable data for synthesis. Random-effects models showed that CCRT was associated with an improvement in overall executive functions for children and adolescents with ADHD (SMD = –0.23, 95% CI: –0.45 to –0.01, *p* = 0.04, *I*^2^ = 31%). Refer to Fig. 4 for more details.

3.4.1.1 Working Memory

**Fig. 5.**
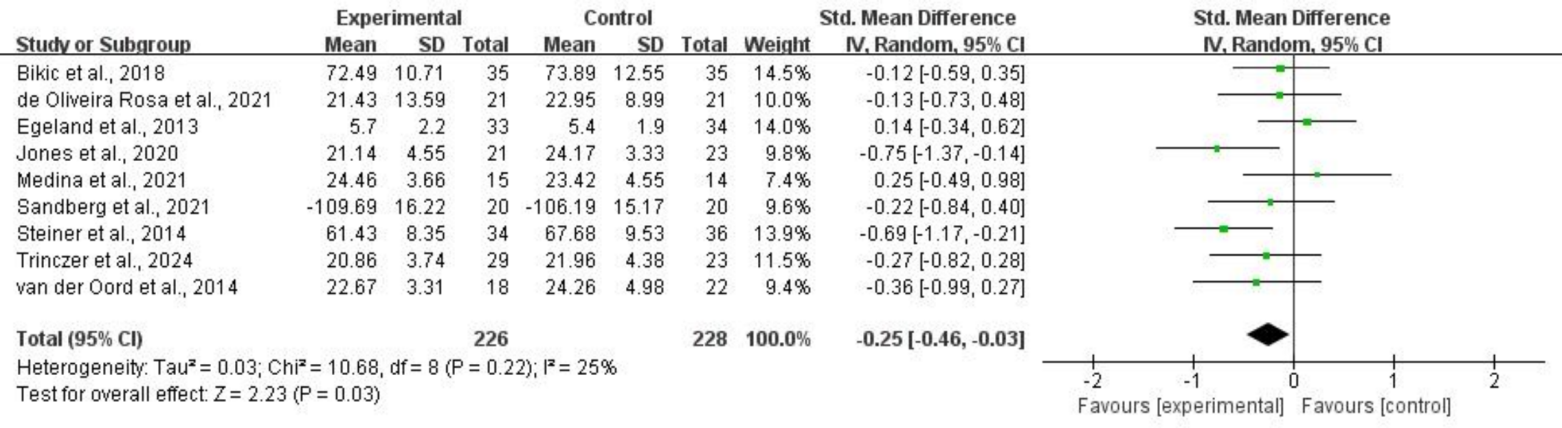
**Working memory**.

For working memory, nine studies comprising 454 subjects provided usable data for synthesis. Random-effects models showed that CCRT was associated with an improvement in working memory for children and adolescents with ADHD (SMD = –0.25, 95% CI: –0.46 to –0.03, *p* = 0.03, *I*^2^ = 25%). Refer to Fig. 5 for more details.

3.4.1.2 Inhibition

**Fig. 6.**
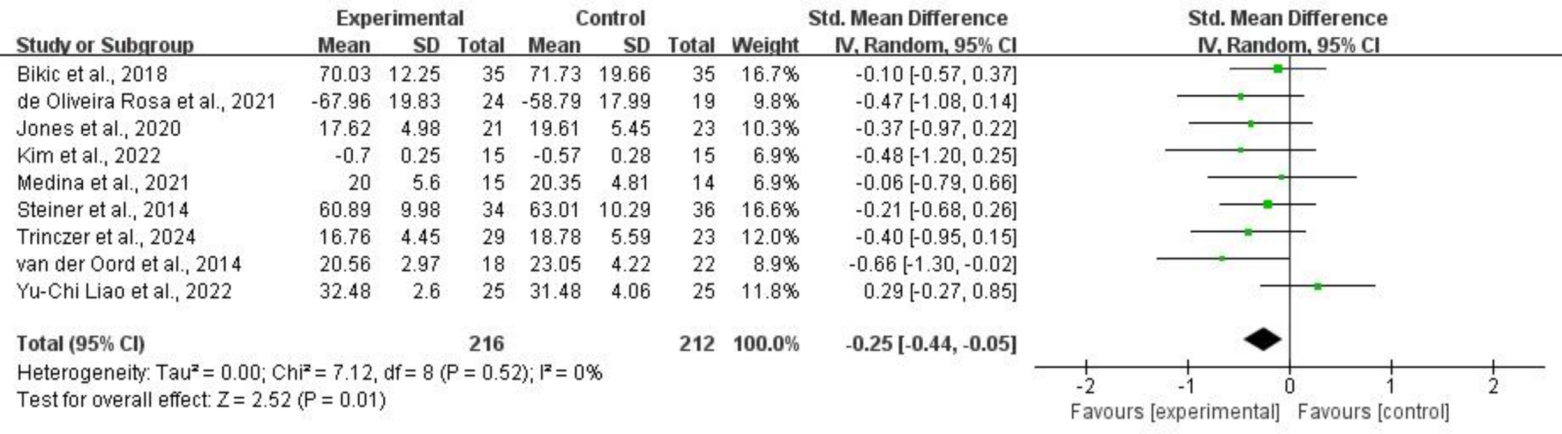
**Inhibition**.

Nine studies, comprising 428 subjects, provided usable data for synthesis on improvements in inhibition. Random-effects models showed that CCRT was associated with a significant improvement in inhibition for children and adolescents with ADHD (SMD = –0.25, 95% CI: –0.44 to –0.05, *p* = 0.01, *I*^2^ = 0%). Refer to Fig. 6 for more details.

3.4.1.3 Cognitive Flexibility

**Fig. 7.**
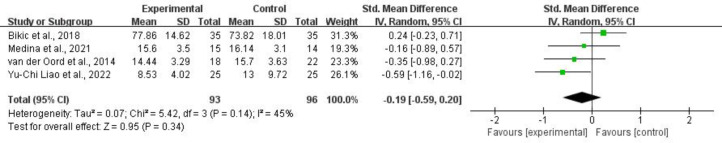
**Cognitive flexibility**.

For cognitive flexibility, four studies comprising 189 subjects provided usable data for synthesis. Random-effects models showed that CCRT did not significantly improve cognitive flexibility for children and adolescents with ADHD (SMD = –0.19, 95% CI: –0.59 to 0.20, *p* = 0.34, *I*^2^ = 45%). Refer to Fig. 7 for more details.

3.4.1.4 Planning

**Fig. 8.**
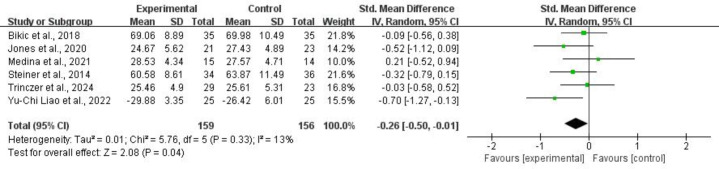
**Planning**.

For planning, six studies comprising 315 subjects provided usable data for synthesis. Random-effects models showed that CCRT was associated with an improvement in planning for children and adolescents with ADHD (SMD = –0.26, 95% CI: –0.50 to –0.01, *p* = 0.04, *I*^2^ = 13%). Refer to Fig. 8 for more details.

3.4.1.5 Emotional Control

**Fig. 9.**
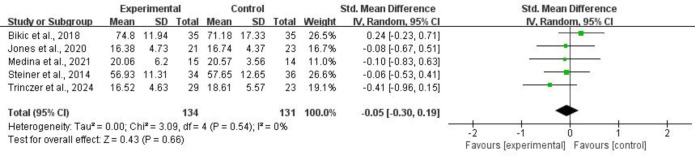
**Emotional control**.

For emotional control, five studies comprising 265 subjects provided usable data for synthesis. Random-effects models showed that CCRT did not significantly improve emotional control for children and adolescents with ADHD (SMD = –0.05, 95% CI: –0.30 to 0.19, *p* = 0.66, *I*^2^ = 0%). Refer to Fig. 9 for more details.

#### 3.4.2 Overall Clinical Symptoms

**Fig. 10.**
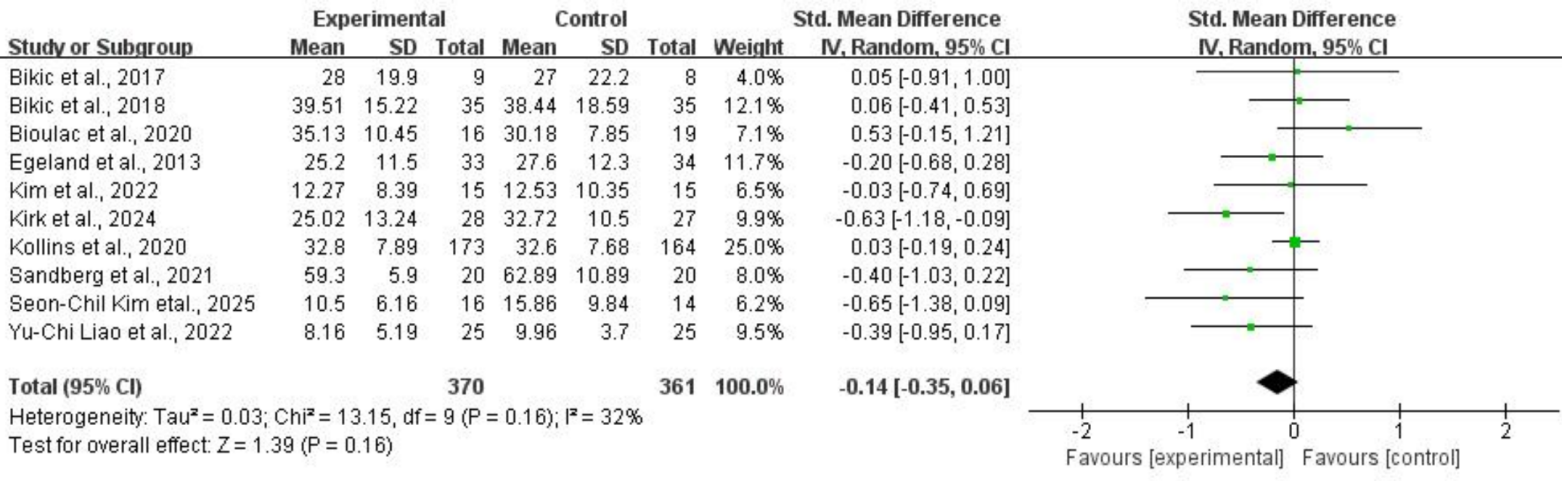
**Overall clinical symptoms**.

For overall clinical symptoms, ten studies comprising 731 subjects provided usable data for synthesis. Random-effects models showed that CCRT did not significantly reduce overall clinical symptoms for children and adolescents with ADHD (SMD = –0.14, 95% CI: –0.35 to 0.06, *p* = 0.16, *I*^2^ = 32%). Refer to Fig. 10 for more details.

3.4.2.1 Inattention

**Fig. 11.**
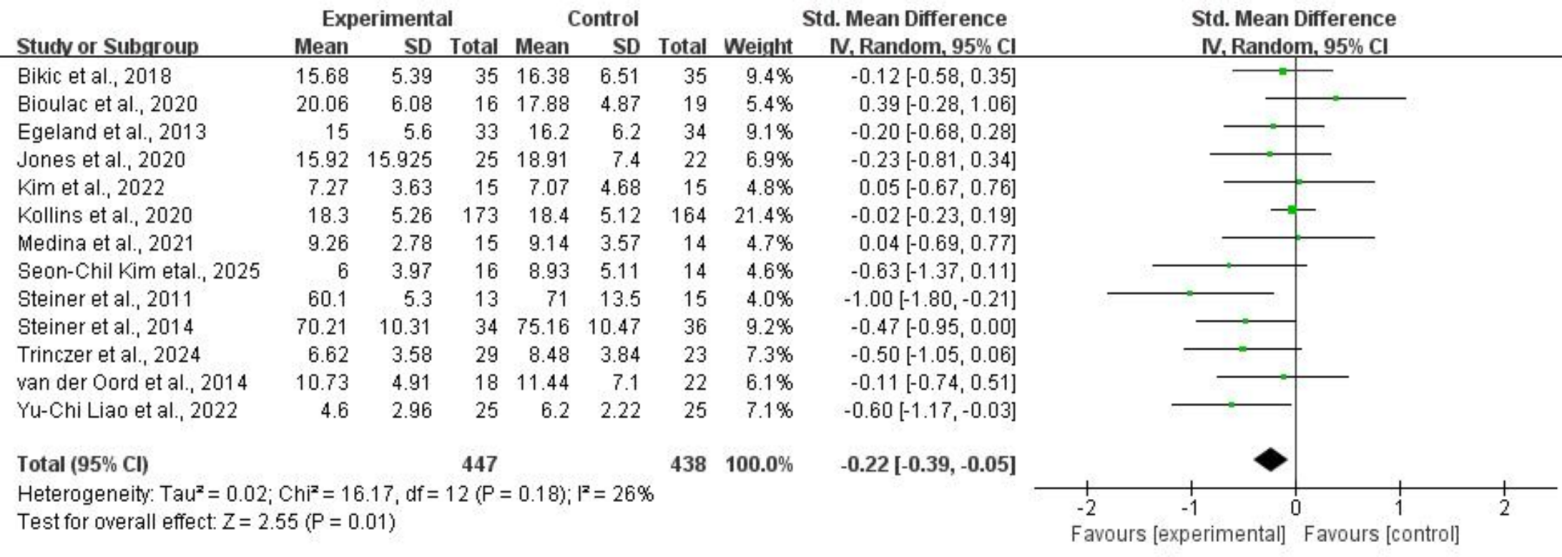
**Inattention**.

Thirteen studies comprising 885 subjects provided usable data on inattention symptoms. Application of a random-effects model showed that CCRT was associated with a reduction in inattention symptoms for children and adolescents with ADHD (SMD = –0.22, 95% CI: –0.39 to –0.05, *p* = 0.01, *I*^2^ = 26%). Refer to Fig. 11 for more details.

3.4.2.2 Hyperactivity/Impulsivity

**Fig. 12.**
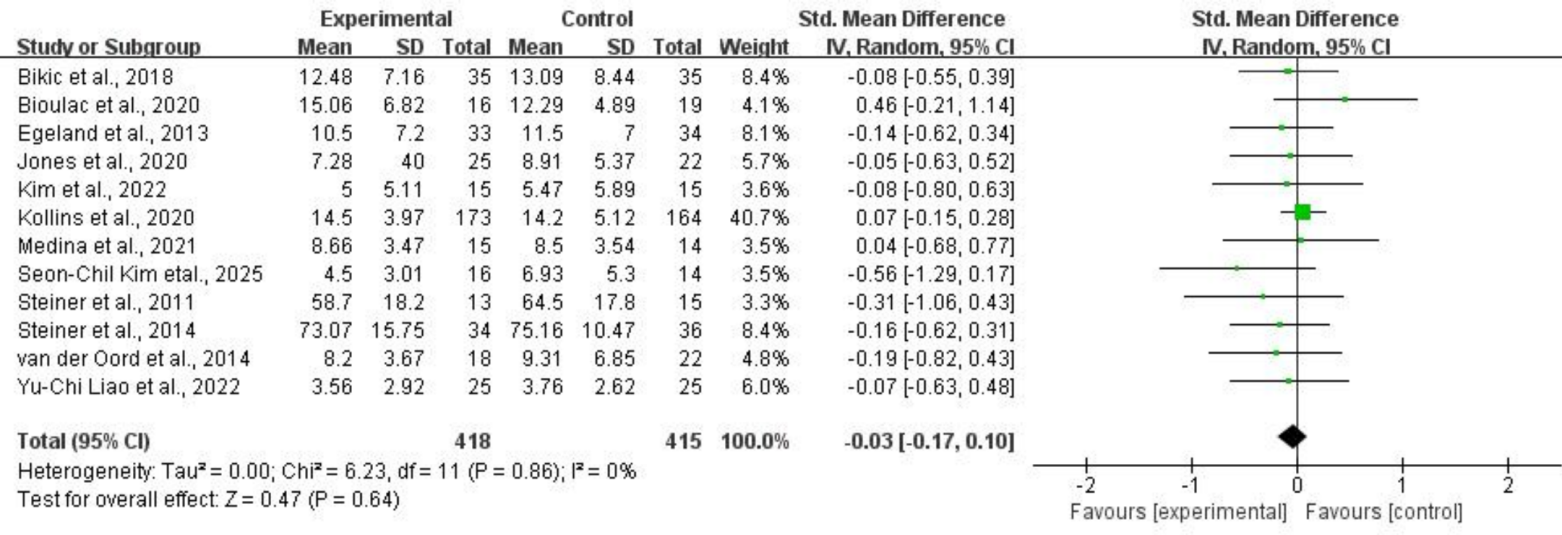
**Hyperactivity/impulsivity**.

For hyperactivity/impulsivity, twelve studies of 833 subjects provided usable data for synthesis. Random-effects models showed that CCRT did not significantly reduce hyperactivity/impulsivity symptoms for children and adolescents with ADHD (SMD = –0.03, 95% CI: –0.17 to 0.10, *p* = 0.64, *I*^2^ = 0%). Refer to Fig. 12 for more details.

### 3.5 Sensitivity Analyses and Publication Bias

All pooled effect sizes remained stable in magnitude and direction when individual studies were removed one at a time. This indicates that the results of the present study were unaffected by the exclusion of any single article, thereby demonstrating the good robustness and stability of the conclusions [37].

There was no evidence of publication bias based on the symmetrical funnel plots for the included studies (see supplementary funnel plots). Similarly, Egger’s test found no significant publication bias concerning overall clinical symptoms (*p* = 0.328), inattention (*p* = 0.145) and hyperactivity/impulsivity (*p* = 0.160).

## 4. Discussion

This meta-analysis synthesizes evidence from 17 randomized controlled trials to evaluate the efficacy of CCRT in overall executive functions, clinical symptoms and their sub-domains in children and adolescents with ADHD. Our findings indicated that CCRT was effective in enhancing overall executive functions in children and adolescents with ADHD. This finding aligned with a previous meta-analysis that reported similar effects of CCRT on executive functioning in ADHD patients across 13 trials [28]. The effectiveness of CCRT may be attributed to the immature development of children’s brains, with better neuroplasticity and greater response to targeted cognitive interventions. In addition, CCRT adjusts the difficulty and content of training based on individual patient differences, thereby better meeting specific needs and effectively improving the executive functions of patients [48]. Furthermore, the gamified nature of many CCRT programs may enhance engagement and compliance, particularly among younger populations. Given its personalized, gamified, and non-invasive nature, CCRT can serve as a beneficial complement to clinical pharmacologic and behavioral interventions in children and adolescents with ADHD. Clinicians may consider incorporating CCRT into multimodal treatment regimens, especially for children with inadequate response or intolerance to drug therapy.

Regarding the sub-dimensions of executive functions, we found notable enhancements in the three core sub-dimensions of working memory, inhibitory control, and planning ability following CCRT. The limited number of systematic reviews exploring the effects of CCRT on multiple sub-dimensions of executive functions among children and adolescents with ADHD presents a challenge in directly comparing our findings to previous studies. Nevertheless, the results of our review could be analyzed within a neurophysiological context: (a) CCRT has been posited to improve executive functions related to working memory by activating specific brain areas, promoting neural plasticity, strengthening neural network connectivity, and optimizing cognitive processing [57, 58]; (b) working memory, inhibition, cognitive flexibility, and planning are interrelated variables within executive functions tasks, each contributing to varying degrees in complex executive tasks [59]. Therefore, the observed effect of CCRT on these sub-dimensions of executive functions likely results from their intricate interaction. Future research should further explore the specific interaction mechanisms among different executive function sub-dimensions during CCRT intervention. Based on these findings, more targeted and individualized intervention modules should be developed to enhance overall executive function of children and adolescents with ADHD and highlight its clinical value.

While CCRT has been demonstrated to improve executive functions, no significant effects have been observed on emotional control and cognitive flexibility. Indeed, enhancing cognitive flexibility may necessitate specific training tasks; however, current CCRT systems predominantly utilize repetitive tasks with fixed rules, such as number matching and response inhibition training, which lack direct stimulation of cognitive transformation ability [59]. Besides, computer-based cognitive training programs primarily target “cold cognition” and lack direct intervention in emotion regulation [60]. Given that only four randomized controlled trials were included to assess the effects of CCRT on cognitive flexibility and emotional control, conclusions derived from our findings should be interpreted with caution. Further studies are warranted to verify the efficacy of CCRT in emotional control and cognitive flexibility.

With regard to clinical symptoms, the present study found that CCRT exhibited a significant effect on inattentive symptoms in children and adolescents with ADHD, but not on overall ADHD symptoms or hyperactivity/impulsivity. This finding was in line with previous research and suggests that CCRT may be particularly effective in enhancing cognitive control and executive functions closely related to attention, resulting in neural changes in brain networks associated with inhibitory control and working memory. These results highlight the utility of CCRT in improving attention-related cognitive control, supporting its preferential application in patients with predominant attention deficits. However, such changes reportedly exhibit limited ability to generate broader improvements in overall ADHD symptoms or hyperactivity/impulsivity domains [28, 61].

Multiple factors account for the limited impact of CCRT on overall ADHD symptoms and hyperactivity/impulsivity. ADHD manifests with inattention, hyperactivity, and impulsivity. Such clinical complexity and heterogeneity present challenges in comprehensively addressing ADHD core symptoms through simple interventions [62]. Consequently, a more personalized and targeted treatment approach is necessary to adequately address the specific cognitive and behavioral profiles of individual patients. Variability in training duration and intensity across included studies may also contribute to non-significant findings. In terms of intervention duration, CCRT treatment over 10 weeks had a significant effect on improvement in inattention symptoms and hyperactivity/impulsivity symptoms [63]. Longer and more intensive courses of CCRT are therefore expected to achieve more durable and clinically significant effects. Beyond treatment duration, age has been identified as a significant moderator of treatment response. Due to brain development and plasticity, younger children exhibit more significant improvement after CCRT [23, 61]. These findings highlight the importance of implementing interventions during early developmental stages.

The neurobiological mechanisms underlying hyperactivity/impulsivity symptoms warrant attention. Hyperactivity and impulsivity are often driven by underlying neurobiological factors that might not be effectively addressed through cognitive training alone [64]. This finding highlights the need for multimodal intervention strategies. Future studies could systematically integrate CCRT and behavioral therapy or individualized drug therapy to simultaneously target the neurobiological basis and behavioral phenotype of symptoms, thereby achieving multi-level intervention goals [65].

Although this review primarily sought to evaluate the effect of CCRT on executive function and clinical symptoms, the presence of a modulatory effect between executive function and clinical symptoms warrants further consideration. Due to the limited number of included studies reporting both types of outcomes and the heterogeneity of measurement instruments, definitive conclusions cannot be drawn at present. Future studies with larger samples, more standardized assessment methods, and longitudinal or mechanistic analyses are needed to explore whether and how changes in executive functioning affect clinical symptoms.

## 5. Limitations

The limitations of this study should be acknowledged. Indeed, our search was limited to English and Chinese databases, which might have resulted in the exclusion of eligible studies published in other languages. Secondly, the quality of the enrolled studies, characterized by a risk of bias and small sample sizes, may affect the reliability of the evidence and limit the generalizability of the results.

## 6. Conclusion

CCRT exhibits selective efficacy in improving specific domains of executive functioning and clinical symptoms in individuals with ADHD. While CCRT enhances overall executive functions, working memory, inhibitory control, and planning abilities in children and adolescents, its effects on cognitive flexibility remain undetermined. Clinically, CCRT effectively reduces inattention symptoms but does not demonstrate significant improvements in overall ADHD symptoms or hyperactivity/impulsivity. Future efforts should focus on developing focused and targeted interventions for each stage of ADHD.

## Availability of Data and Materials

The datasets for this study are available from the corresponding author on reasonable request.

## Supplementary Material

Supplementary material associated with this article can be found, in the online version, at https://doi.org/10.31083/AP39972.
