# Supplementary material for: Effectiveness of Computerized Cognitive Remediation Therapy on Executive Functions and Clinical Symptoms in Children and Adolescents With ADHD: A Meta-Analysis of Randomized Controlled Trials
Source: Alpha Psychiatry. 2026 Mar 17;27(2):39972. doi: 10.31083/AP39972 (PMC13156065; doi:10.31083/AP39972)
Supplement: Supplementary file 1 [file 2757-8038-27-2-39972-s1.zip › Supplementary Material.docx]

**PRISMA2020 checklist**

| **Section and Topic** | **Item #** | **Checklist item** | **Location where item is reported** |
| --- | --- | --- | --- |
| **TITLE** | | |  |
| Title | 1 | Identify the report as a systematic review. | 1 |
| **ABSTRACT** | | |  |
| Abstract | 2 | See the PRISMA 2020 for Abstracts checklist. | 1 |
| **INTRODUCTION** | | |  |
| Rationale | 3 | Describe the rationale for the review in the context of existing knowledge. | 1-2 |
| Objectives | 4 | Provide an explicit statement of the objective(s) or question(s) the review addresses. | 2-3 |
| **METHODS** | | |  |
| Eligibility criteria | 5 | Specify the inclusion and exclusion criteria for the review and how studies were grouped for the syntheses. | 3 |
| Information sources | 6 | Specify all databases, registers, websites, organisations, reference lists and other sources searched or consulted to identify studies. Specify the date when each source was last searched or consulted. | 3 |
| Search strategy | 7 | Present the full search strategies for all databases, registers and websites, including any filters and limits used. | Supplementary Material |
| Selection process | 8 | Specify the methods used to decide whether a study met the inclusion criteria of the review, including how many reviewers screened each record and each report retrieved, whether they worked independently, and if applicable, details of automation tools used in the process. | 3 |
| Data collection process | 9 | Specify the methods used to collect data from reports, including how many reviewers collected data from each report, whether they worked independently, any processes for obtaining or confirming data from study investigators, and if applicable, details of automation tools used in the process. | 3 |
| Data items | 10a | List and define all outcomes for which data were sought. Specify whether all results that were compatible with each outcome domain in each study were sought (e.g. for all measures, time points, analyses), and if not, the methods used to decide which results to collect. | 3 |
|  | 10b | List and define all other variables for which data were sought (e.g. participant and intervention characteristics, funding sources). Describe any assumptions made about any missing or unclear information. | 3 |
| Study risk of bias assessment | 11 | Specify the methods used to assess risk of bias in the included studies, including details of the tool(s) used, how many reviewers assessed each study and whether they worked independently, and if applicable, details of automation tools used in the process. | 3 |
| Effect measures | 12 | Specify for each outcome the effect measure(s) (e.g. risk ratio, mean difference) used in the synthesis or presentation of results. | 8 |
| Synthesis methods | 13a | Describe the processes used to decide which studies were eligible for each synthesis (e.g. tabulating the study intervention characteristics and comparing against the planned groups for each synthesis (item #5)). | 8 |
|  | 13b | Describe any methods required to prepare the data for presentation or synthesis, such as handling of missing summary statistics, or data conversions. | 8 |
|  | 13c | Describe any methods used to tabulate or visually display results of individual studies and syntheses. | 8 |
|  | 13d | Describe any methods used to synthesize results and provide a rationale for the choice(s). If meta-analysis was performed, describe the model(s), method(s) to identify the presence and extent of statistical heterogeneity, and software package(s) used. | 8 |
|  | 13e | Describe any methods used to explore possible causes of heterogeneity among study results (e.g. subgroup analysis, meta-regression). | 8 |
|  | 13f | Describe any sensitivity analyses conducted to assess robustness of the synthesized results. | 8 |
| Reporting bias assessment | 14 | Describe any methods used to assess risk of bias due to missing results in a synthesis (arising from reporting biases). | 8 |
| Certainty assessment | 15 | Describe any methods used to assess certainty (or confidence) in the body of evidence for an outcome. | 8 |
| **RESULTS** | | |  |
| Study selection | 16a | Describe the results of the search and selection process, from the number of records identified in the search to the number of studies included in the review, ideally using a flow diagram. | 8-9 |
|  | 16b | Cite studies that might appear to meet the inclusion criteria, but which were excluded, and explain why they were excluded. | 8-9 |
| Study characteristics | 17 | Cite each included study and present its characteristics. | 4-8 |
| Risk of bias in studies | 18 | Present assessments of risk of bias for each included study. | 4-8,10 |
| Results of individual studies | 19 | For all outcomes, present, for each study: (a) summary statistics for each group (where appropriate) and (b) an effect estimate and its precision (e.g. confidence/credible interval), ideally using structured tables or plots. | 8-13 |
| Results of syntheses | 20a | For each synthesis, briefly summarise the characteristics and risk of bias among contributing studies. | 8-13 |
|  | 20b | Present results of all statistical syntheses conducted. If meta-analysis was done, present for each the summary estimate and its precision (e.g. confidence/credible interval) and measures of statistical heterogeneity. If comparing groups, describe the direction of the effect. | 8-13 |
|  | 20c | Present results of all investigations of possible causes of heterogeneity among study results. | None |
|  | 20d | Present results of all sensitivity analyses conducted to assess the robustness of the synthesized results. | 11 |
| Reporting biases | 21 | Present assessments of risk of bias due to missing results (arising from reporting biases) for each synthesis assessed. | 11 |
| Certainty of evidence | 22 | Present assessments of certainty (or confidence) in the body of evidence for each outcome assessed. | 8-13 |
| **DISCUSSION** | | |  |
| Discussion | 23a | Provide a general interpretation of the results in the context of other evidence. | 13-14 |
|  | 23b | Discuss any limitations of the evidence included in the review. | 13-14 |
|  | 23c | Discuss any limitations of the review processes used. | 13-14 |
|  | 23d | Discuss implications of the results for practice, policy, and future research. | 13-14 |
| **OTHER INFORMATION** | | |  |
| Registration and protocol | 24a | Provide registration information for the review, including register name and registration number, or state that the review was not registered. | 1，3 |
|  | 24b | Indicate where the review protocol can be accessed, or state that a protocol was not prepared. | ,3 |
|  | 24c | Describe and explain any amendments to information provided at registration or in the protocol. | None |
| Support | 25 | Describe sources of financial or non-financial support for the review, and the role of the funders or sponsors in the review. | 15 |
| Competing interests | 26 | Declare any competing interests of review authors. | 15 |
| Availability of data, code and other materials | 27 | Report which of the following are publicly available and where they can be found: template data collection forms; data extracted from included studies; data used for all analyses; analytic code; any other materials used in the review. | 14 |

*From:*  Page MJ, McKenzie JE, Bossuyt PM, Boutron I, Hoffmann TC, Mulrow CD, et al. The PRISMA 2020 statement: an updated guideline for reporting systematic reviews. BMJ 2021;372:n71. doi: 10.1136/bmj.n71. This work is licensed under CC BY 4.0. To view a copy of this license, visit <https://creativecommons.org/licenses/by/4.0/>

**Search Strategy**

- Search dates: up until 05/25/2025

Limits: language (English and Chinese)

| **1.PubMed** | | |
| --- | --- | --- |
| procedure | Search mode | Number of outcomes |
| #1 | (((((Virtual reality[MeSH Terms]) OR (computer[MeSH Terms])) OR (Mobile Applications[MeSH Terms])) OR (Therapy, Computer-Assisted[MeSH Terms])) OR (Internet[MeSH Terms])) OR (Digital Technology[MeSH Terms]) OR (Telemedicine[MeSH Terms]) OR (Digital health[MeSH Terms]) |  |
| #2 | digit*[Title/Abstract] OR VR[Title/Abstract] OR Virtual reality[Title/Abstract] OR Internet[Title/Abstract] OR Therapy, Computer-Assisted[Title/Abstract] OR Mobile Application*[Title/Abstract] OR ehealth[Title/Abstract] OR online*[Title/Abstract] OR webs*[Title/Abstract] OR tablet*[Title/Abstract] OR comput*[Title/Abstract] OR Telemedicine[Title/Abstract] OR Telemonitoring[Title/Abstract] OR mhealth[Title/Abstract] |  |
| #3 | #1 OR #2 |  |
| #4 | (Cognitive Behavioral Therapy[MeSH Terms]) OR (Cognitive Remediation[MeSH Terms]) |  |
| #5 | cognitive remediation[Title/Abstract] OR ICBT[Title/Abstract] OR CCBT[Title/Abstract] OR ECBT[Title/Abstract] OR cognitive psychotherap*[Title/Abstract] OR cognit* therap*[Title/Abstract] OR behavio* therap*[Title/Abstract] OR cognit* behavio* therap*[Title/Abstract] |  |
| #6 | #4 OR #5 |  |
| #7 | Attention Deficit Disorder with Hyperactivity[MeSH Terms] |  |
| #8 | ADHD[Title/Abstract] OR ADDH[Title/Abstract] OR Attention Deficit Disorder*[Title/Abstract] OR Hyperkinetic Syndrome[Title/Abstract] OR Minimal Brain Dysfunction[Title/Abstract] OR Attention Deficit Disorder with Hyperactivity[Title/Abstract] |  |
| #9 | #7 OR #8 |  |
| #10 | #3 AND #6 AND #9 | 128 |

| **2.web of science（Core collection）** | | |
| --- | --- | --- |
| procedure | Search mode | Number of outcomes |
| #1 | TS=(comput* OR Internet OR computer assisted therapy OR Mobile Application* OR Virtual reality OR digit* OR VR OR ehealth OR online* OR webs* OR tablet* OR Telemedicine OR telemonitoring OR mhealth) |  |
| #2 | TS=(cognit* behavio* therap* OR cognitive remediation OR ICBT OR CCBT OR ECBT OR cognitive psychotherap* OR cognit* therap* OR behavio* therap* ) |  |
| #3 | TS=(Attention Deficit Disorder with Hyperactivity OR ADHD OR ADDH OR Attention Deficit Disorder* OR Deficit-Hyperactivity Disder*, Attention OR Hyperkinetic Syndrome OR Minimal Brain Dysfunction ) |  |
| #4 | #3 AND #2 AND #1 | 449 |

| **3.embase** | | |
| --- | --- | --- |
| procedure | Search mode | Number of outcomes |
| #1 | computer'/exp OR 'internet'/exp OR 'computer assisted therapy'/exp OR 'mobile application'/exp OR 'virtual reality'/exp OR 'digital technology'/exp OR 'Telemedicine'/exp OR 'Digital health'/exp |  |
| #2 | comput*':ab,ti OR 'internet':ab,ti OR 'computer assisted therapy':ab,ti OR 'mobile application*':ab,ti OR 'virtual reality':ab,ti OR 'digit*':ab,ti OR 'VR':ab,ti OR 'ehealth':ab,ti OR 'online*':ab,ti OR 'webs*':ab,ti OR 'tablet*':ab,ti OR 'Telemedicine':ab,ti OR 'Telemonitoring':ab,ti OR 'mhealth':ab,ti |  |
| #3 | #1 OR #2 |  |
| #4 | cognitive behavioral therapy'/exp OR 'cognitive remediation therapy'/exp |  |
| #5 | cognit* behavio* therap*':ab,ti OR 'cognitive remediation':ab,ti OR 'icbt':ab,ti OR 'ccbt':ab,ti OR 'ecbt':ab,ti OR 'cognitive psychotherap*':ab,ti OR 'cognit* therap*':ab,ti OR 'behavio* therap*':ab,ti |  |
| #6 | #4 OR #5 |  |
| #7 | attention deficit hyperactivity disorder'/exp |  |
| #8 | attention deficit disorder with hyperactivity':ab,ti OR 'adhd':ab,ti OR 'addh':ab,ti OR 'attention deficit disorder*':ab,ti OR 'deficit-hyperactivity disder*, attention':ab,ti OR 'hyperkinetic syndrome':ab,ti OR 'minimal brain dysfunction':ab,ti |  |
| #9 | #7 OR #8 |  |
| #10 | #3 AND #6 AND #9 | 403 |

| **4.Cochrane Library** | | |  |
| --- | --- | --- | --- |
| procedure | Search mode | Number of outcomes |  |
| #1 | MeSH descriptor: [Virtual Reality] explode all trees |  |  |
| #2 | MeSH descriptor: [Computers] explode all trees |  |  |
| #3 | MeSH descriptor: [Mobile Applications] explode all trees |  |  |
| #4 | MeSH descriptor: [Therapy, Computer-Assisted] explode all trees |  |  |
| #5 | MeSH descriptor: [Internet] explode all trees |  |  |
| #6 | MeSH descriptor: [Telemedicine] explode all trees |  |  |
| #7 | MeSH descriptor: [Digital Technology] explode all trees |  |  |
| #8 | (digit* OR VR OR Virtual reality OR Internet OR Therapy, Computer-Assisted OR Mobile Application* OR ehealth OR online* OR webs* OR tablet* OR comput* OR Telemedicine OR mhealth):ti,ab,kw |  |  |
| #9 | #1 OR #2 OR #3 OR #4 OR #5 OR #6 OR #7 OR #8 |  |  |
| #10 | MeSH descriptor: [Cognitive Behavioral Therapy] explode all trees |  |  |
| #11 | MeSH descriptor: [Cognitive Remediation] explode all trees |  |  |
| #12 | | (cognitive remediation OR ICBT OR CCBT OR ECBT OR cognitive psychotherap* OR cognit* therap* OR behavio* therap* OR cognit* behavio* therap*):ti,ab,kw |  |
| #13 | | #10 OR #11 OR #12 |  |
| #14 | | MeSH descriptor: [Attention Deficit Disorder with Hyperactivity] explode all trees |  |
| #15 | | (ADHD OR ADDH OR Attention Deficit NEXT Disorder* OR Deficit-Hyperactivity NEXT Disder*, Attention OR Hyperkinetic Syndrome OR Minimal Brain Dysfunction):ti,ab,kw |  |
| #16 | | #14 OR #15 |  |
| #17 | | #9 AND #13 AND #16 | 455 |

| **5.PsycINFO** | | |
| --- | --- | --- |
| procedure | Search mode | Number of outcomes |
| #1 | MA ( Internet OR computer assisted therapy OR Mobile Applications OR Virtual reality OR Digital Technology OR Telemedicine ) OR TI ( comput* OR Internet OR computer assisted therapy OR Mobile Application* OR Virtual reality OR digit* OR VR OR ehealth OR online* OR webs* OR tablet* ) OR AB ( comput* OR Internet OR computer assisted therapy OR Mobile Application* OR Virtual reality OR digit* OR VR OR ehealth OR online* OR webs* OR tablet* OR Telemedicine OR mHealth OR telemonitoring ) |  |
| #2 | MA Cognitive Remediation OR TI ( cognit* behavio* therap* OR cognitive remediation OR ICBT OR CCBT OR ECBT OR cognitive psychotherap* OR cognit* therap* OR behavio* therap* ) OR AB ( cognit* behavio* therap* OR cognitive remediation OR ICBT OR CCBT OR ECBT OR cognitive psychotherap* OR cognit* therap* OR behavio* therap* ) |  |
| #3 | MA Attention Deficit Disorder with Hyperactivity OR TI ( Attention Deficit Disorder with Hyperactivity OR ADHD OR ADDH OR Attention Deficit Disorder* OR Deficit-Hyperactivity Disder*, Attention OR Hyperkinetic Syndrome OR Minimal Brain Dysfunction ) OR AB ( Attention Deficit Disorder with Hyperactivity OR ADHD OR ADDH OR Attention Deficit Disorder* OR Deficit-Hyperactivity Disder*, Attention OR Hyperkinetic Syndrome OR Minimal Brain Dysfunction ) |  |
| #4 | #1 AND #2 AND #3 | 244 |

| **6.CBM** | | |
| --- | --- | --- |
| procedure | Search mode | Number of outcomes |
| #1 | ( "虚拟现实"[不加权:扩展] OR "计算机"[不加权:扩展] OR "移动应用"[不加权:扩展] OR "治疗，计算机辅助"[不加权:扩展] OR "因特网"[不加权:扩展] OR "数字技术"[不加权:扩展] OR "远程医疗”[不加权:扩展]) |  |
| #2 | ( "数字化"[常用字段:智能] OR "VR"[常用字段:智能] OR "虚拟现实"[常用字段:智能] OR "计算机"[常用字段:智能] OR "移动应用"[常用字段:智能] OR "网络"[常用字段:智能] OR "CCRT"[常用字段:智能] OR "因特网"[常用字段:智能] OR "互联网"[常用字段:智能] OR "计算机辅助治疗"[常用字段:智能] OR "治疗，计算机辅助"[常用字段:智能] OR "APP"[常用字段:智能] OR "应用程序"[常用字段:智能] OR "数字技术"[常用字段:智能] OR "远程"[常用字段:智能] OR "mhealth"[常用字段:智能]) |  |
| #3 | #1 OR #2 |  |
| #4 | "认知行为疗法"[不加权:扩展] OR "认知矫正"[不加权:扩展] |  |
| #5 | ( "认知干预"[常用字段:智能] OR "认知训练"[常用字段:智能] OR "认知矫正"[常用字段:智能] OR "认知行为疗法"[常用字段:智能] OR "认知治疗"[常用字段:智能]) |  |
| #6 | #4 OR #5 |  |
| #7 | "注意力缺陷障碍伴多动"[不加权:扩展] |  |
| #8 | ( "ADHD"[常用字段:智能] OR "ADDH"[常用字段:智能] OR "多动症"[常用字段:智能] OR "多动综合征"[常用字段:智能] OR "注意力缺陷障碍伴多动"[常用字段:智能] OR "注意力缺陷"[常用字段:智能]) |  |
| #9 | #7 OR #8 |  |
| #10 | #3 AND #6 AND #9 | 8 |

| **7.CNKI** | | |
| --- | --- | --- |
| procedure | Search mode | Number of outcomes |
| #1 | 篇关摘：数字化 + VR + 虚拟现实 + 计算机 + 移动应用 + 网络 + 因特网 + 互联网 + 计算机辅助治疗 + 治疗，计算机辅助 + APP + 应用程序 + 数字技术 + 远程医疗 + mhealth + ehealth |  |
| #2 | 篇关摘：认知干预 + 认知训练 + 认知矫正 + 认知行为疗法 + 认知治疗 |  |
| #3 | 篇关摘：ADHD + ADDH + 多动症 + 多动综合征 + 注意力缺陷障碍伴多动 + 注意力缺陷 |  |
| #4 | #1 AND #2 AND #3 | 34 |
| 注：勾选了“同义词扩展” | | |

| **8.WANFANG** | | |
| --- | --- | --- |
| procedure | Search mode | Number of outcomes |
| #1 | 主题：数字化 OR VR OR 虚拟现实 OR 计算机 OR 移动应用 OR 网络 OR 因特网 OR 互联网 OR 计算机辅助治疗 OR 治疗，计算机辅助 OR APP OR 应用程序 OR 数字技术 OR 远程 OR mhealth OR ehealth |  |
| #2 | 主题：认知干预 OR 认知训练 OR 认知矫正 OR 认知行为疗法 OR 认知治疗 |  |
| #3 | 主题：ADHD OR ADDH OR 多动症 OR 多动综合征 OR 注意力缺陷障碍伴多动 OR 注意力缺陷 |  |
| #4 | #1 AND #2 AND #3 | 102 |
| 注：勾选了“同义词扩展” | | |

| **9.VIP** | | |
| --- | --- | --- |
| procedure | Search mode | Number of outcomes |
| #1 | 摘要：数字化 OR VR OR 虚拟现实 OR 计算机 OR 移动应用 OR 网络 OR 因特网 OR 互联网 OR 计算机辅助治疗 OR 治疗，计算机辅助 OR APP OR 应用程序 OR 数字技术 OR 远程 OR mhealth OR ehealth |  |
| #2 | 摘要：认知干预 OR 认知训练 OR 认知矫正 OR 认知行为疗法 OR 认知治疗 |  |
| #3 | 摘要：ADHD OR ADDH OR 多动症 OR 多动综合征 OR 注意力缺陷障碍伴多动 OR 注意力缺陷 |  |
| #4 | #1 AND #2 AND #3 | 21 |

**Supplementary Table GRADE analyses**

| **outcomes** | **N(studies)** | **Risk of bias** | **Inconsistency** | **Indirectness** | **Imprecision** | **Publication bias** | **Overall quality of evidence** |
| --- | --- | --- | --- | --- | --- | --- | --- |
| **overall executive function** | 500(9) | Serious^1^ | No | No | No | Undetected | ⊝⊕⊕⊕ **moderate** |
| **working memory** | 454(9) | Serious^1^ | No | No | No | Undetected | ⊝⊕⊕⊕ **moderate** |
| **inhibition** | 428(9) | Serious^1^ | No | No | No | Undetected | ⊝⊕⊕⊕ **moderate** |
| **cognitive flexibility** | 189(4) | Serious^1^ | Serious^2^ | No | No | Undetected | ⊝⊝⊕⊕ **low** |
| **Planning** | 315(6) | No | Serious^2^ | No | No | Undetected | ⊝⊕⊕⊕ **moderate** |
| **Emotional Control** | 265(5) | Serious^1^ | Serious^2^ | No | No | Undetected | ⊝⊝⊕⊕ **low** |
| **overall clinical symptoms** | 731(10) | No | No | No | No | Undetected | ⊕⊕⊕⊕ **high** |
| **Inattention** | 885(13) | No | No | No | No | Undetected | ⊕⊕⊕⊕ **high** |
| **Hyperactivity/Impulsivity** | 833(13) | No | No | No | No | Undetected | ⊕⊕⊕⊕ **high** |
| GRADE Working Group grades of evidence **High quality:** Further research is very unlikely to change our confidence in the estimate of effect.  **Moderate quality:** Further research is likely to have an important impact on our confidence in the estimate of effect and may change the estimate. **Low quality:** Further research is very likely to have an important impact on our confidence in the estimate of effect and is likely to change the estimate. **Very low quality:** We are very uncertain about the estimate. | | | | | | | |
| ^1^ The proportion of high-risk studies was high. ^2^ For continuous outcomes, N<400. | | | | | | | |

**
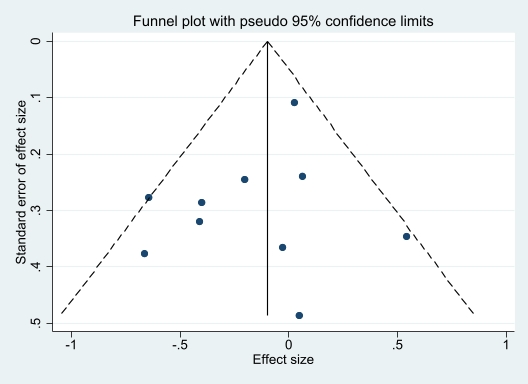
**

**Overall clinical symptoms funnel plot**

**
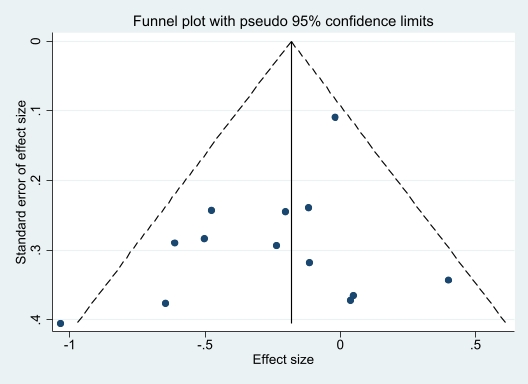
**

**Inattention funnel plot**

**
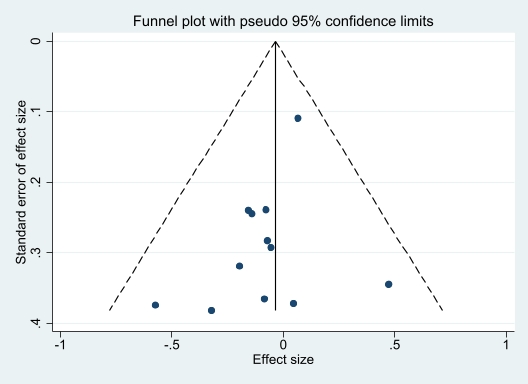
**

**Hyperactivity/impulsivity funnel plot**
